# Clinical, Immunohistochemical, and Inflammatory Profiles in Colorectal Cancer: The Impact of MMR Deficiency

**DOI:** 10.3390/diagnostics15172141

**Published:** 2025-08-25

**Authors:** Vlad Alexandru Ionescu, Gina Gheorghe, Ioana Alexandra Baban, Alexandru Barbu, Ninel Iacobus Antonie, Teodor Florin Georgescu, Razvan Matei Bratu, Carmen Cristina Diaconu, Cristina Mambet, Coralia Bleotu, Valentin Enache, Camelia Cristina Diaconu

**Affiliations:** 1 Faculty of Medicine, University of Medicine and Pharmacy Carol Davila Bucharest, 050474 Bucharest, Romania; vladalexandru.ionescu92@gmail.com (V.A.I.); ioana.baban99@gmail.com (I.A.B.); alexandru.barbu212@gmail.com (A.B.); ninel-iacobus@drd.umfcd.ro (N.I.A.); florin.georgescu1@yahoo.com (T.F.G.); razvan.bratu@umfcd.ro (R.M.B.); cristina.mambet@gmail.com (C.M.); camelia.diaconu@umfcd.ro (C.C.D.); 2 Internal Medicine Department, Clinical Emergency Hospital of Bucharest, 105402 Bucharest, Romania; 3 General Surgery Department, Clinical Emergency Hospital of Bucharest, 105402 Bucharest, Romania; 4 Department of Cellular and Molecular Pathology, Stefan S. Nicolau Institute of Virology, Romanian Academy, 030304 Bucharest, Romania; carmen.diaconu@virology.ro (C.C.D.); cbleotu@yahoo.com (C.B.); 5 Hematology Department, Emergency University Clinical Hospital of Bucharest, 050098 Bucharest, Romania; 6 Research Institute of the University of Bucharest (ICUB), University of Bucharest, 060023 Bucharest, Romania; 7 Academy of Romanian Scientists, 050085 Bucharest, Romania; 8 Department of Anatomical Pathology, Clinical Emergency Hospital of Bucharest, 105402 Bucharest, Romania; valienache00@gmail.com

**Keywords:** colorectal cancer, mismatch repair deficiency, CDX2, neutrophil-to-lymphocyte ratio, platelet-to-lymphocyte ratio

## Abstract

**Background/Objectives:** Mismatch repair (MMR) deficiency assessment has proven to be a valuable tool for prognostic evaluation and therapeutic management guidance in patients with colorectal cancer (CRC). Our study aimed to investigate the associations between MMR deficiency and a range of clinicopathological parameters. **Methods:** We conducted a retrospective observational study including 264 patients diagnosed with CRC, for whom immunohistochemical (IHC) data were available. Statistical analysis was performed using the Python 3.12.7 programming language within the Jupyter Notebook environment (Anaconda distribution). **Results:** MMR deficiency was identified in 18.18% of patients. It was significantly associated with younger age (<50 years), female sex, right-sided tumor location, poor tumor differentiation (G3), smoking, and loss of CDX2 expression (*p* < 0.001). MLH1 and PMS2 were the most frequently affected proteins, with concurrent loss in 77.08% of MMR-deficient cases. Loss of MLH1 expression correlated with female sex (*p* = 0.004), right-sided location (*p* < 0.001), poor differentiation (*p* < 0.001), and loss of CDX2 expression (*p* < 0.001). Additionally, the loss of PMS2 expression was associated with female sex (*p* = 0.015), right-sided tumor location (*p* = 0.003), and poor differentiation (*p* < 0.001). No significant associations were identified between MMR status and tumor stage, histological subtype, PLR, or NLR values. **Conclusions:** Gaining deeper insights into the clinical relevance of MMR status in CRC could contribute to improved testing rates and support the design of tailored management strategies that address the specific biological features of these tumors.

## 1. Introduction

Colorectal cancer (CRC) is currently the third most prevalent malignancy and the second leading cause of cancer-related death worldwide, with approximately 1.9 million new cases and 0.9 million deaths reported annually [1,2]. The continuous evolution of therapeutic regimens and the growing understanding of CRC pathogenesis underscore the need for personalized management of these patients. Risk stratification based solely on tumor stage and histological grade often proves insufficient for accurately predicting therapeutic response or individual prognosis [3].

In recent years, the assessment of microsatellite instability (MSI) and mismatch repair (MMR) deficiency has emerged as an important tool for determining treatment response and prognosis in CRC patients [3]. Microsatellites are short, repetitive DNA sequences prone to replication errors, scattered throughout the genome [4]. The mismatch repair system, involving proteins such as MLH1 (mutL homolog 1), MSH2 (mutS homolog 2), MSH6 (mutS homolog 6), and PMS2 (postmeiotic segregation increased 2), plays a critical role in correcting these replication errors [4]. MSI, caused by MMR deficiency or DNA polymerase errors, is implicated in the development of CRC [4]. MMR deficiency refers to the impaired function or loss of one or more proteins involved in the DNA mismatch repair mechanism. In contrast, MSI represents the downstream biological consequence of this dysfunction, namely, the accumulation of insertion or deletion mutations in microsatellite regions throughout the genome. MSI serves as a hallmark of Lynch syndrome but is also observed in approximately 10–20% of sporadic CRC cases, frequently associated with epigenetic silencing of the *MLH1* gene [5,6,7]. Mutations in *MLH1* or *MSH2* genes confer a significantly increased lifetime risk of cancer (70–80%), whereas mutations in MSH6 or PMS2 are associated with lower risk (25–60%) [8]. Furthermore, unlike Lynch syndrome, sporadic MSI is often associated with the *BRAFV600E* mutation (in approximately 50% of cases) and a more aggressive phenotype [9,10].

MSI colorectal tumors often present with distinct clinicopathological features, including proximal location, advanced tumor stage, poor differentiation, and prominent lymphocytic infiltration [11]. However, the prognostic role of MSI in CRC remains controversial. Several studies have shown that patients with MSI-positive CRC exhibit more favorable outcomes compared to those with microsatellite-stable (MSS) tumors [12,13], possibly due to a more robust antitumor immune response [11]. Mohan et al. reported better disease-free survival among patients with stage I–II MSI CRC compared to those with MSS tumors [14]. In contrast, among stage III patients, MSI tumors were associated with increased lymphovascular and perineural invasion, as well as shorter disease-free survival relative to MSS tumors at the same stage [14].

In the past decades, increasing attention has been directed toward the role of host immune response in tumor progression [11]. MSI has been associated with elevated expression of several immune checkpoint molecules, including programmed cell death protein 1 (PD-1), programmed death-ligand 1 (PD-L1), and cytotoxic T-lymphocyte-associated antigen 4 (CTLA-4) [11]. This has led to the hypothesis that immune checkpoint blockade may be particularly effective in this subgroup of patients, supporting a molecularly-driven therapeutic approach independent of tumor location or histology [11]. As a result, in 2017, the U.S. Food and Drug Administration (FDA) approved pembrolizumab, a PD-1 inhibitor, for patients with unresectable or metastatic MSI-high solid tumors, regardless of origin or histological type [15].

The optimal management of CRC patients requires a thorough understanding of the molecular mechanisms underlying tumorigenesis [16]. This knowledge may also contribute to the identification of prognostic biomarkers capable of predicting disease progression [16]. Among such markers, the neutrophil-to-lymphocyte ratio (NLR) and platelet-to-lymphocyte ratio (PLR) have recently been proposed, as their values may reflect immune response alterations in CRC patients [16,17,18]. The systemic inflammatory response to tumors has been associated with changes in several hematologic parameters, especially neutrophils and lymphocytes [16]. Moreover, various hypotheses have been proposed to explain the relationship between elevated platelet counts and cancer progression [16]. Platelets release proangiogenic and growth factors, including platelet factor 4 (PF4), platelet-derived growth factor (PDGF), and transforming growth factor-beta (TGF-β), all of which promote tumor progression [16].

Although the clinical significance of NLR is not yet fully understood, it has been suggested that an elevated NLR may indicate a shift toward a proinflammatory immune profile (reflected by higher neutrophil counts) and diminished cellular immunity (reflected by lower lymphocyte counts) [16]. Since platelets are key mediators of inflammation, both thrombocytosis and elevated PLR are likely part of the same pathophysiological process. Notably, both NLR and PLR have been correlated with tumor stage and differentiation grade in CRC [16,17,18].

The primary objective of this study was to evaluate the MMR status of CRC patients and to investigate its correlation with clinical, immunohistochemical, and inflammatory features. A secondary aim was to identify prognostic biomarkers with potential relevance for therapeutic decision-making and survival outcomes.

## 2. Materials and Methods

We conducted a retrospective, observational study over a 10-year period (January 2015–January 2025), which included 264 patients hospitalized at the Emergency Clinical Hospital in Bucharest with a histopathologically confirmed diagnosis of colorectal cancer (CRC), for whom immunohistochemistry (IHC) results were available. We divided the patients into two groups: Group 1 included 216 patients with a positive MMR status (defined as preserved expression of all four MMR proteins), while Group 2 comprised 48 patients with a negative MMR status (defined as loss of expression of at least one MMR protein).

This study received approval from the Ethics Committee of the Emergency Clinical Hospital, Bucharest (Approval No. 1400/07.02.2023). All patients included in this study signed an informed consent form agreeing to the use of their personal data for medical education and research purposes.

**Figure 1:**
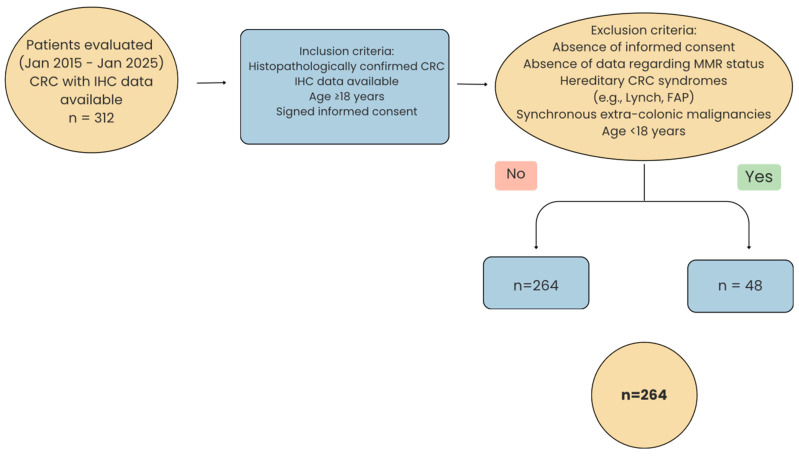
Flowchart of patient selection according to inclusion and exclusion criteria (FAP—familial adenomatous polyposis).

The inclusion criteria were as follows: histopathologically confirmed colorectal cancer, availability of immunohistochemical (IHC) results, age ≥ 18 years, and signed informed consent. Exclusion criteria included the following: absence of informed consent, lack of data regarding MMR status, known hereditary colorectal cancer syndromes (e.g., Lynch syndrome, FAP—familial adenomatous polyposis), synchronous extra-colonic malignancies, and patients younger than 18 years. Accordingly, for 48 of the patients initially enrolled in this study, MMR status data were not available; these cases were therefore excluded, yielding a final cohort of 264 patients (Figure 1).

The study database was compiled by reviewing patient observation charts along with pathological, imaging, and laboratory investigation results, as archived and digitally stored in the hospital’s information system, according to the predefined inclusion and exclusion criteria. The variables analyzed included age, sex, smoking status, alcohol consumption, comorbidities, tumor location, endoscopic appearance, histological type, degree of differentiation, TNM stage, the expression of immunohistochemical markers [MLH1, MSH2, MSH6, PMS2, Ki67 (Kiel 67 antigen), CK20 (cytokeratin 20), CK7 (cytokeratin 7), and CDX2 (caudal-type homeobox 2)], as well as systemic inflammatory markers calculated from preoperative blood counts (NLR, PLR), length of hospitalization, and in-hospital mortality.

Immunohistochemical (IHC) staining was performed using the following primary antibodies: MLH1 (clone G168-728, dilution 1:100), MSH2 (clone ZR260, dilution 1:200), MSH6 (clone ZR342, dilution 1:200), PMS2 (clone ZR317, dilution 1:200), CK20 (clone ZM42, dilution 1:200), CDX2 (clone ZR215, dilution 1:200), CK7 (clone ZM2067MT, dilution 1:200), and Ki67 (clone ZM2305MT, dilution 1:300). Nuclear staining in tumor cells was considered positive for MLH1, MSH2, MSH6, and PMS2, provided that stromal or lymphoid cells served as internal positive controls. Loss of nuclear staining in tumor cells in the presence of positive internal controls was interpreted as negative. Ki67 expression was evaluated as the percentage of tumor cell nuclei showing positive staining. CK20 and CK 7 were considered positive when cytoplasmatic staining was present in ≥10% of tumor cells. Diffuse and strong nuclear staining of CDX2 is evidence of colorectal origin. Comorbidities were defined according to standard criteria: obesity, as BMI ≥ 30 kg/m^2^; hypertension, as blood pressure ≥ 140/90 mmHg or ongoing antihypertensive therapy; dyslipidemia, as total cholesterol ≥ 240 mg/dL, LDL ≥ 160 mg/dL, triglycerides ≥ 200 mg/dL, or the use of lipid-lowering drugs; and diabetes mellitus, as fasting glucose ≥ 126 mg/dL, HbA1c ≥ 6.5%, or ongoing antidiabetic therapy. All patients underwent either curative or palliative surgical interventions; thus, histological type, tumor grade, stage, and immunohistochemical status were determined from surgically excised tumor specimens. Additionally, the NLR and PLR values were calculated using complete blood counts obtained at the time of hospital admission, prior to any surgical intervention. The selection of immunohistochemical and inflammatory markers was based exclusively on their availability within the database of the Emergency Clinical Hospital.

### Statistical Analysis

Data analysis was performed using the Python 3.12.7 programming language in the Jupyter Notebook environment (Anaconda distribution). Categorical variables were reported as absolute frequencies and percentages. The distribution of continuous variables was assessed using the Shapiro–Wilk test and visually confirmed through histograms and Q-Q plots where applicable. Based on the normality of distribution, comparisons between groups were performed using the independent samples Student’s t-test (for normally distributed variables) or the Mann–Whitney U test (for non-parametric distributions).

For the Ki67 proliferation index, we used a cutoff value of 25%, in accordance with the recommendations of the American Joint Committee on Cancer (AJCC) [19].

Categorical variables were analyzed using the chi-square (χ^2^) test or Fisher’s exact test, depending on the distribution of observed frequencies. Associations between the studied variables were explored both descriptively and through inferential methods. To assess the strength of association between categorical variables, Cramér’s V coefficient was employed.

To evaluate the influence of predictive variables on relevant clinicopathological outcomes, we applied both univariate and multivariate linear regression models, validated using the bootstrap method (1000 iterations). The robustness of regression coefficients was assessed by calculating 95% confidence intervals (95% CI) for each predictor included in the model. Additionally, to estimate the relative impact of clinical and biological variables on tumor characteristics, we implemented Random Forest Regressor models, complemented by SHapley Additive exPlanations (SHAP) values to interpret the contribution of each predictor to the variation in the outcome.

The threshold for statistical significance was set at *p* < 0.05. All results were interpreted in a clinical context, aiming to identify potential prognostic correlations relevant to patient outcomes.

## 3. Results

Immunohistochemical evaluation was available for all patients included in our study; however, there was no complete consistency in the panel of markers assessed (Table 1). MMR protein expression was evaluated in all patients included in our study, while the Ki67 proliferation index was assessed in 39% of them. A deficiency in at least one MMR protein was observed in 18.18% of the patients included in our study (Table 1). Among these, 6.25% had a deficiency in a single MMR protein, 91.67% had a deficiency in two proteins, and 2.08% (corresponding to a single case) showed loss of all four MMR proteins (Table 2). Among the MMR components, the highest rate of loss of expression was observed for MLH1, followed by PMS2 (Table 1). Concurrent loss of both MLH1 and PMS2 was identified in 77.08% of patients with MMR deficiency (Table 2). Regarding Ki67 expression, the vast majority of patients (97.08%) exhibited values greater than 25% (Table 1).

We initially conducted a descriptive analysis of the study cohort. Regarding age group distribution, patients with a positive MMR status were predominantly in the 60–70-year age group, whereas those with a negative MMR status were primarily under the age of 50 (Table 3). However, these differences were not statistically significant (*p* = 0.853). In terms of sex distribution, loss of expression of at least one MMR protein was more frequently observed in female patients, whereas preserved expression of all MMR proteins (MMR-positive status) was significantly associated with male sex, a difference that reached statistical significance (Table 3).

Another parameter assessed in our study was the anatomical location of the tumors and their endoscopic appearance. We observed a predominance of left-sided colon tumors in the group of patients with a positive MMR status, whereas right-sided tumor localization was more frequent among patients with a negative MMR status. This difference was statistically significant (Table 3). Regarding endoscopic appearance, stenosing tumors were predominant in both groups (Table 3).

We subsequently performed a comparative evaluation between the two patient groups regarding tumor histologic subtype, tumor differentiation grade, and TNM staging (Table 3). In both groups, adenocarcinomas not otherwise specified (NOS) were predominant. Although mucinous adenocarcinomas were more frequently observed in Group 2 compared to Group 1 (14.58% vs. 9.26%), this difference was not statistically significant (Table 3). Similarly, no statistically significant differences were identified between the two groups in terms of TNM stage, with stage IIA being the most prevalent in both groups, followed closely by stage IIIB (Table 3). Regarding histological grade, patients in Group 1 more frequently presented with moderately differentiated (G2) tumors (66.20%), whereas poorly differentiated (G3) tumors were more common among patients in Group 2 (62.5%), this difference being statistically significant (Table 3).

In regard to the most common comorbidities, the only statistically significant difference we identified was in smoking, which was reported more frequently by patients with a negative MMR status compared to those with a positive MMR status (Table 3).

Data on additional immunohistochemical markers beyond MMR protein expression were available for a limited number of patients. Among these, the only statistically significant difference between the two groups was observed in CDX2 expression, with loss of CDX2 more frequently associated with loss of MMR expression (Table 3).

The only systemic inflammatory markers available for all patients included in this study were the neutrophil-to-lymphocyte ratio (NLR) and the platelet-to-lymphocyte ratio (PLR) (Table 3). The levels of these inflammatory biomarkers did not show significant differences between patients with a positive MMR status and those with a negative MMM status (Table 3).

We further investigated the correlation between tumor location and histological type, as well as between tumor location and grade of differentiation (Table 4). The contingency table analysis showed a relatively uniform distribution of tumor histological types across all intestinal segments. The chi-square test yielded a value of χ^2^ = 8.38 (df = 9), with a significance level of *p* = 0.4966, indicating no statistically significant association between tumor histological type and anatomical location (Table 4). The observed frequencies were very close to those expected under the assumption of independence, and the Cramér’s V value of 0 indicated a negligible practical effect (Table 4). Thus, in this dataset, the proportions of each histological subtype remained consistent regardless of the affected intestinal segment, without evidence of preferential distribution.

In contrast, the analysis of the association between tumor location and grade of differentiation revealed a statistically significant relationship. The chi-square test result was χ^2^ = 46.02 (df = 6), with a significance level of *p* < 0.001 and a small-to-moderate effect size (Cramér’s V = 0.255) (Table 4). Poorly differentiated tumors (G3) were significantly overrepresented in the right colon compared to expectations, while moderately differentiated tumors (G2) predominated in the left colon and rectum (Table 4). Well-differentiated tumors (G1) were more frequently observed in the rectum compared to the overall average. The expected frequencies, under the assumption of independence, were exceeded in the cells corresponding to these associations, confirming that the distribution of differentiation grades is not random. Although the absolute number of synchronous tumors was low, a higher-than-expected frequency of G1 and G2 differentiation grades was observed in this subgroup (Table 4).

Overall, these findings suggest the existence of distinct biological or clinical patterns depending on the affected colonic segment, with potential implications for tumor pathogenesis, staging, therapeutic decision-making, and patient prognosis.

Consistent with these observations, we found that the MMR-deficient tumors in our cohort were predominantly located in the right colon and were more frequently poorly differentiated. Nevertheless, our analysis also revealed that, irrespective of MMR status, right-sided tumors overall tended to exhibit a higher proportion of poor differentiation compared to left-sided or rectal tumors. This suggests that the association between MMR deficiency and poor differentiation may, at least in part, be driven by the intrinsic distribution pattern of differentiation grades across anatomical subsites.

In the next stage, we assessed potential correlations between the expression of each MMR protein and several variables, including age, sex, tumor location, endoscopic appearance, histological type, differentiation grade, tumor stage, the expression of other immunohistochemical markers (Ki67, CK7, CK20, CDX2), and systemic inflammatory markers (NLR and PLR).

Loss of MLH1 expression was significantly more frequent in female patients compared to males (21.1% vs. 8.9%, *p* = 0.004), in tumors located in the right colon (28.4% in right colon vs. 10.4% in left colon and 6.2% in rectum, *p* < 0.001), and in poorly differentiated tumors (0% negative MLH1 status in G1, 8.3% in G2, and 26.6% in G3, *p* < 0.001). Male patients exhibited an approximately threefold lower risk of MLH1 loss of expression compared to females. Moreover, the loss of MSH1 expression was significantly associated with the loss of CDX2 expression (*p* < 0.001). Regarding tumor stage, although the proportion of MLH1-negative cases ranged from 2.8% in stage I to 17.3% in stage IIIC, these differences were not statistically significant (*p* = 0.632). Similarly, MLH1 positive expression varied from 0% in neuroendocrine tumors to 16.1% in mucinous adenocarcinomas, without reaching statistical significance (*p* = 0.196). No correlation was identified between MLH1 expression and tumor endoscopic morphology (*p* = 0.630). Furthermore, no significant associations were observed between MLH1 expression and CK7 expression (*p* = 1.00), CK20 expression (*p* = 1.00), or the Ki67 proliferation index (*p* = 1.00). With respect to systemic inflammation assessed through NLR and PLR, no statistically significant correlations were found between these markers and MLH1 status.

Loss of MSH2 expression was rare (≤3.6%) and was significantly associated only with poorly differentiated tumors (*p* = 0.01). Although signet-ring cell adenocarcinomas tended to exhibit a higher frequency of MSH2 loss, this trend did not reach statistical significance (*p* = 0.063).

Similarly, loss of MSH6 expression was also rare (<3%), with the only statistically significant correlation being with histological type. MSH6 loss was predominantly observed in signet-ring cell adenocarcinomas and mucinous adenocarcinomas. Specifically, MSH6-negative status was found in 20% of signet-ring cell adenocarcinomas, 6.5% of mucinous adenocarcinomas, 0.8% of adenocarcinomas NOS, and 0% of neuroendocrine tumors (*p* = 0.001).

PMS2 loss was significantly associated with female sex (18% vs. 8.4%, *p* = 0.015), right-sided tumor location (23.5% in right colon, 8.8% in left colon, and 7.2% in rectum; *p* = 0.003), and poor differentiation (24.8% in G3, 6.7% in G2, and 0% in G1; *p* < 0.001). Thus, females had approximately twice the likelihood of exhibiting PMS2 loss compared to males. Additionally, PMS2-negative status was more frequently observed in signet-ring cell adenocarcinomas (20%) and mucinous adenocarcinomas (16.1%) compared to NOS adenocarcinomas (12.5%) or neuroendocrine tumors (0%), though these differences were not statistically significant (*p* = 0.531). No statistically significant correlations were identified between PMS2 expression loss and endoscopic tumor appearance (*p* = 0.662), tumor stage (*p* = 0.92), Ki-67 proliferation index (*p* = 1.00), CK7 expression loss (*p* = 1.00), CK20 expression loss (*p* = 0.797), CDX2 expression loss (*p* = 0.505), NLR (*p* = 0.271), or PLR (*p* = 0.079). However, we observed a tendency toward higher PLR values in patients with PMS2-negative status, which may suggest a potential biological association.

The mean duration of hospitalization was 13.9 days, with a minimum of 1 day and a maximum of 58 days. The in-hospital mortality rate was 7.69%. We subsequently explored the determinants of hospitalization duration and in-hospital mortality using multivariate linear regression models. Initially, the target variable was the duration of hospitalization, followed by in-hospital mortality, with the following predictors: tumor stage; grade of differentiation; anatomical location; and the expression status of MLH1, MSH2, MSH6, and PMS2. To estimate confidence intervals, we applied the bootstrap method, with 1000 resamplings (B = 1000).

To further identify the variables with the greatest influence on hospitalization duration and in-hospital mortality, we implemented a Random Forest Regressor using the same set of explanatory variables. We then applied SHapley Additive exPlanations (SHAP) analysis to quantify the contribution of each predictor variable.

**Figure 2:**
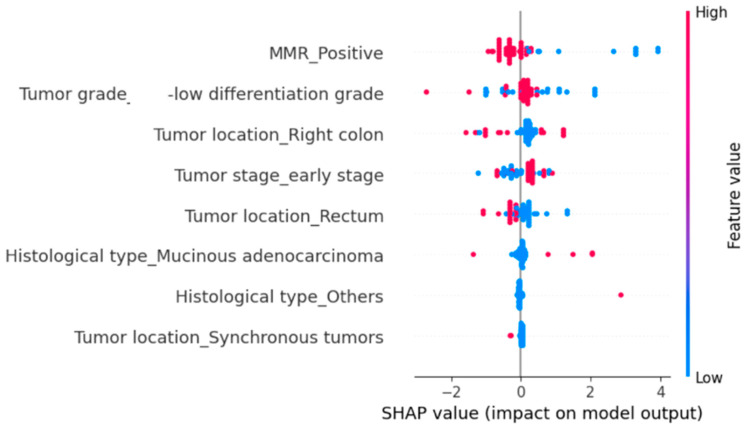
Influence of clinicopathological variables on hospital stay duration estimated using SHAP values in a Random Forest Regressor Model. Each dot represents an individual patient, and the color gradient indicates the original feature value (red = high; blue = low). Variables on the *y*-axis are ordered by overall importance.

The SHAP analysis indicated that MMR positivity and poor tumor differentiation (G3) were the strongest predictors of prolonged hospitalization (Figure 2). Right-sided tumor location also contributed to increased hospitalization length, although with a more modest effect. In contrast, early-stage tumors, rectal location, mucinous adenocarcinoma, and other histological subtypes (e.g., signet-ring cell carcinoma, neuroendocrine tumors) were generally associated with shorter hospital stays. Synchronous tumors were infrequent but, in selected cases, showed a marked positive impact on hospitalization length (Figure 2).

**Figure 3:**
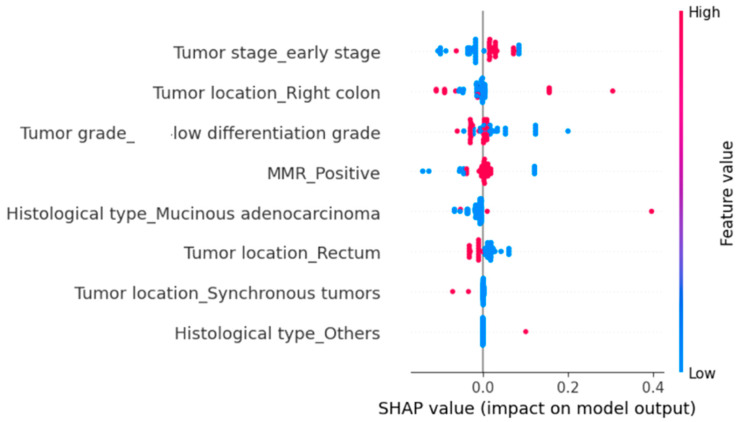
Influence of clinicopathological variables on in-hospital mortality rate, estimated using SHAP values in a Random Forest Regressor Model. Higher SHAP values indicate a greater positive contribution to the predicted in-hospital mortality. Each dot represents a patient; color reflects the relative value of the variable (blue = low; red = high).

Regarding in-hospital mortality, mucinous adenocarcinoma proved to be the most influential parameter, significantly increasing the risk of death (Figure 3). Right-sided tumor location and poor differentiation grade also tended to shift the predictions to the right, thereby raising the probability of mortality. Early-stage disease and MMR positivity showed smaller, generally positive effects (Figure 3). In contrast, rectal location and other histological subtypes, such as signet-ring cell carcinoma or neuroendocrine tumors, usually shifted the predictions to the left, reducing the probability of death (Figure 3). Synchronous tumors exhibited minimal influence, clustering around zero (Figure 3). In conclusion, the effects of these predictors on in-hospital mortality were modest (most SHAP values < 0.1–0.2, with isolated cases up to 0.4), indicating that the model’s predictions result from the cumulative contribution of multiple small factors rather than from a single dominant determinant.

**Figure 4:**
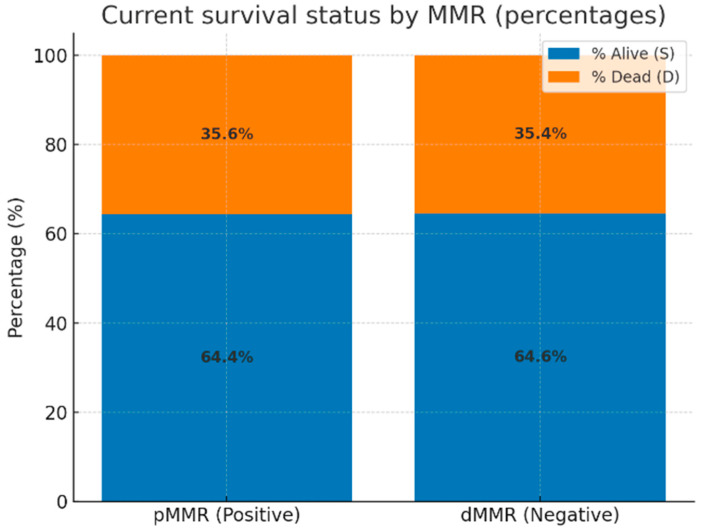
Current survival status stratified by MMR status (orange = deceased; blue = alive).

We further evaluated the current survival status of all 264 patients stratified by MMR status. As illustrated in Figure 4, the proportion of deceased patients was similar between the two groups: 35.6% in the proficient MMR (pMMR) cohort and 35.4% in the deficient MMR (dMMR) cohort. Accordingly, no statistically significant difference in survival status was observed between MMR-proficient and MMR-deficient tumors (*p* = 1.00; odds ratio = 0.99; 95% CI: 0.51–1.90).

**Figure 5:**
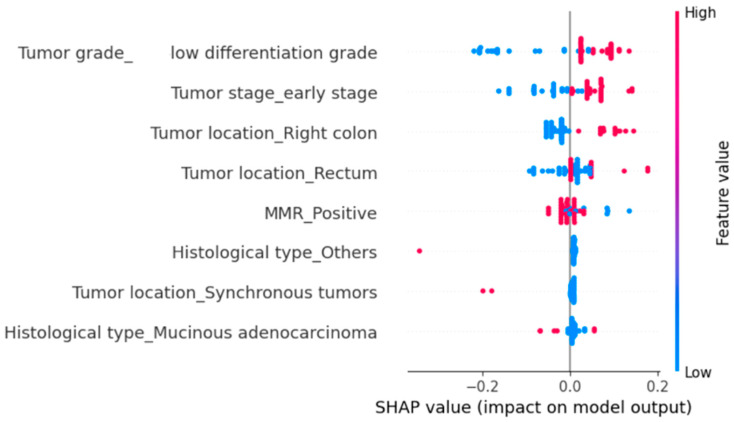
Influence of clinicopathological variables on current survival status estimated using SHAP values in a Random Forest Regressor model. Positive SHAP values indicate a greater contribution to the predicted probability of in-hospital mortality, while negative values indicate a reduced probability. Each dot represents an individual patient; the color reflects the relative value of the variable (blue = low; red = high).

We subsequently evaluated the impact of several clinicopathological parameters on all-cause mortality using SHAP analysis (Figure 5). The parameters significantly associated with an increased risk of death were poor tumor differentiation and right-sided tumor location (Figure 5). MMR positivity also increased the probability of mortality, although the effect was more modest (Figure 5). Synchronous tumors were rare, and their overall contribution was limited, yet they tended to increase the risk of death (Figure 5). In contrast, early-stage disease and rectal tumor location were associated with a reduced probability of mortality (Figure 5).

## 4. Discussion

MMR deficiency was identified in 18.18% of the patients included in our study. Among these cases, 6.25% exhibited isolated loss of a single MMR protein, 91.67% showed concurrent loss of two proteins, and 2.08% (a single patient) demonstrated complete loss of all four MMR proteins. Among the individual components, MLH1 showed the highest frequency of loss, followed by PMS2. Notably, concurrent loss of MLH1 and PMS2 was observed in 77.08% of patients with MMR deficiency.

Despite advancements in the diagnostic and therapeutic management of CRC, this malignancy remains one of the most pressing global public health issues. The implementation of CRC screening strategies has improved early detection rates in individuals over the age of 50, contributing to a subsequent reduction in disease-related morbidity and mortality [20]. However, in recent decades, an alarming increase has been observed in CRC cases diagnosed before the age of 50, now classified as early-onset colorectal cancer (EO-CRC) [21]. In our study, although the 60–70-year age group was predominant among patients with a positive MMR status (32.87%), the proportion of patients under 50 years of age was higher in the group with a negative MMR status (25%). These findings are consistent with previously published data, which report that MMR deficiency is frequently observed in patients with EO-CRC [21,22].

Regarding sex distribution, we observed a predominance of male patients in the group with positive MMR status compared to a predominance of female patients in the group with negative MMR status (62.55% vs. 36.1%), a difference that was statistically significant (*p* < 0.001). These findings are in line with existing literature, which reports a higher prevalence of MMR deficiency among female patients [23]. Smoking was also more prevalent in Group 2 compared to Group 1. Yang et al. suggested a synergistic effect between smoking and α1-antitrypsin alleles in the development of microsatellite instability–high (MSI-H) CRC [24].

We also assessed the comorbidities of the patients included in our cohort and found that arterial hypertension was present in a considerable proportion of patients in both groups (59.26% in Group 1 and 58.33% in group 2). These findings align with published data indicating hypertension as one of the most common comorbidities among CRC patients [25,26]. A potential explanation for this may lie in the high prevalence of hypertension in the general adult population. Furthermore, our data highlight the significant burden of metabolic comorbidities in the study population. Metabolic reprogramming has been described as a hallmark of cancer, implicated in both tumorigenesis and disease progression [27]. These results underscore the importance of preventive strategies aimed at controlling modifiable risk factors such as overweight and obesity.

Next, we evaluated the anatomical, macroscopic, and histological characteristics of the tumors. We observed a predominance of left-sided colon tumors in Group 1 (43.05%), in contrast to a predominance of right-sided colon tumors in Group 2 (54.17%), a difference that was statistically significant (*p* < 0.001). This is consistent with the findings of Topdagi et al., who reported that 83.9% of tumors in a cohort of 752 CRC patients were left-sided, while 16.1% were located in the right colon [28]. The larger discrepancy compared with our data is likely due to the inclusion of rectal tumors in the left-sided group in that study, whereas in our analysis, rectal tumors were classified separately. On the other hand, Gutiérrez et al., in a study including 101,259 patients with CRC, reported an association between MMR deficiency and tumors located in the right colon, particularly in the ascending colon [29].

We further explored potential associations between tumor location and histological type or differentiation grade. A significant association was observed between right-sided tumors and poor differentiation, and between left-sided or rectal tumors and moderate differentiation. Our findings are in line with previous studies highlighting clinically relevant differences between right- and left-sided CRCs, which are partly explained by their distinct embryological origin, vascularization, and exposure to luminal factors [30,31,32]. Taken together, these data suggest that the higher prevalence of poor differentiation among right-sided tumors in our cohort may reflect inherent biological characteristics of proximal colorectal cancers, independent of MMR status. This observation reinforces the interpretation that while MMR deficiency is strongly associated with right-sided and poorly differentiated tumors, part of this correlation may be driven by the intrinsic clinicopathological profile of right-sided CRCs.

Histologically, the most frequent subtype was adenocarcinoma NOS, in line with literature reports [33]. Regarding tumor grade, poorly differentiated (G3) tumors were predominant in the group of patients with MMR deficiency, compared to moderately differentiated (G2) tumors in the group with positive MMR status. These differences were statistically significant (*p* < 0.001).

As previously mentioned, IHC analysis was available for all patients, which was one of our main inclusion criteria. A key limitation of our study, however, was the incomplete consistency regarding the markers evaluated. MMR protein expression was evaluated in all patients included in our study, while the Ki67 proliferation index was concurrently assessed in a substantial proportion of cases. Other markers, such as CK7, CK20, and CDX2, were available in only a small subset of patients, explaining the lack of statistical significance observed for these variables. Among the aforementioned biomarkers, the only statistically significant correlation identified was between MMR deficiency and the loss of CDX2 expression. CDX2 is a transcription factor essential for the development and differentiation of intestinal epithelial cells. The link between MMR deficiency and CDX2 loss is not yet fully understood; however, some studies suggest that epigenetic modifications, such as DNA methylation, may be involved [34]. Moreover, mutations in the *BRAF* gene, which are frequently implicated in colorectal carcinogenesis, have also been associated with reduced CDX2 expression [35].

We further specifically analyzed the correlations between the loss of expression of each individual MMR protein and clinicopathological variables. Importantly, current international guidelines, including those of the National Comprehensive Cancer Network (NCCN) and the European Society for Medical Oncology (ESMO), recommend the separate immunohistochemical evaluation of MLH1, PMS2, MSH2, and MSH6 in colorectal cancer [36,37]. Although these proteins function biologically as heterodimeric units, their expression loss is not always symmetrical [36,37]. For example, the loss of MSH2 and/or MSH6 expression indicates a suspicion of Lynch syndrome, whereas the loss of MLH1 and PMS2 expression requires further investigation to assess the presence of a BRAF mutation or hypermethylation of the MLH1 gene promoter region [37]. The identification of either of the latter two abnormalities strongly suggests the presence of an acquired somatic alteration of the MLH1 gene rather than Lynch syndrome [37]. These patterns carry distinct diagnostic and prognostic implications, particularly for Lynch syndrome screening and treatment stratification. Therefore, analyzing each protein individually is not only consistent with current standards of care but also provides additional insights into clinicopathological and prognostic correlations, as demonstrated in our cohort.

Loss of MLH1 and PMS2 expression was more common in female patients, poorly differentiated tumors, and right-sided locations. Moreover, the loss of MLH1 expression was significantly correlated with the loss of CDX2 expression (*p* < 0.001); however, this association was not observed with PMS2 expression. Loss of MSH2 and MSH6 expression was observed in only ~3% of patients, which partly explains the lack of significant correlations. Nonetheless, loss of MSH2 was significantly associated with poor tumor differentiation (*p* = 0.01), and loss of MSH6 expression was more frequently observed in patients with signet-ring cell adenocarcinomas and mucinous adenocarcinomas, compared to those with NOS adenocarcinomas (*p* = 0.001). We did not identify any statistically significant correlations between MMR status and the Ki67 proliferation index, NLR, or PLR values.

Our findings are indirectly supported by numerous studies. MSI in sporadic CRC is most frequently related to MLH1 loss, often accompanied by PMS2 loss [11,38,39]. This phenotype results either from a germline mutation in the *MLH1* gene or from acquired somatic hypermethylation of the MLH1 promoter [11]. Germline mutations in *MSH6* and *MSH2* are usually associated with isolated loss of MSH6 and MSH2 protein expression [40]. Ho et al. also reported an association between MSI, female sex, and right-sided CRC [39]. In addition, other studies have linked MSI to mucinous and poorly differentiated adenocarcinomas [40]. Our findings confirm the statistically significant association between MMR deficiency and poor differentiation; however, no significant correlation was observed between histological subtype and MMR status in our cohort.

Regarding prognosis, multiple studies support a favorable correlation between MSI and both overall and disease-free survival in patients with sporadic CRC [39,40]. MSI tumors are characterized by high infiltration of CD8+ and CD4+ T cells, robust antitumor immune responses, and elevated mutational burdens generating numerous neoantigens [41]. These features render MSI tumors particularly responsive to immune checkpoint inhibitors (ICI) [41,42]. In our study, positive expression of MMR proteins was associated with both longer hospitalization and an increased probability of in-hospital death or all-cause mortality, which was evaluated based on the current survival status. Thus, we indirectly confirm the favorable prognostic impact of MMR deficiency.

Beyond confirming previously reported associations between dMMR and younger age, female sex, right-sided tumor location, and poor differentiation, our study provides several additional contributions. First, to our knowledge, this is the first analysis in a Romanian CRC cohort specifically addressing the clinical, immunohistochemical, and inflammatory characteristics of dMMR. Given the underrepresentation of Eastern European populations in molecular oncology research, these results expand the geographical and epidemiological landscape of MMR-deficient CRC. Another novel aspect of our study lies in the differential analysis of each individual MMR protein in relation to a spectrum of clinical characteristics, immunohistochemical markers, and the inflammatory profile (assessed through NLR and PLR) in patients with sporadic CRC. While MMR proteins function biologically as units, their immunohistochemical evaluation and clinical interpretation are performed individually, and differences in expression patterns may carry distinct diagnostic and prognostic significance. This individual-level analysis provides novel insights and complements existing knowledge, offering potential clinical and prognostic implications, particularly in an underrepresented Eastern European population. Although our findings did not reveal significant associations between dMMR and systemic inflammatory markers (NLR and PLR), this negative result remains relevant. Recent studies have proposed NLR and PLR as universal prognostic markers in CRC; however, our results suggest that their predictive value may be population-dependent and not directly related to MMR status. This observation may guide future research toward identifying more specific inflammatory biomarkers linked to DNA repair pathways. In addition, our study demonstrated the association between MMR deficiency and loss of CDX2 expression, an association that remains incompletely elucidated. We therefore consider this finding to represent another important element that may guide future research.

The main limitation of our study was the relatively limited number of immunohistochemical markers evaluated beyond the MMR proteins, as well as the absence of a uniform assessment of these markers across all patients. This inconsistency is partly explained by the extended 10-year period of data collection, during which clinical practice, diagnostic standards, and IHC interpretation methods have evolved. Another important limitation is the inclusion of different histological subtypes (adenocarcinoma NOS, mucinous adenocarcinoma, signet-ring cell adenocarcinoma, and neuroendocrine tumors). These tumor types exhibit heterogeneous biological behavior and distinct sensitivities to chemotherapy, which may have influenced the results and should be taken into account when interpreting our findings. A further limitation of our study is the lack of data regarding the long-term outcomes of the included patients, as they were referred to oncology clinics in other medical centers. While we were able to verify the current survival status for all patients, this limitation, together with the retrospective design, did not allow for a systematic assessment of survival duration or determination of the exact cause of death.

## 5. Conclusions

Our study provides a comprehensive analysis of clinicopathological, immunohistochemical, and inflammatory features in patients with colorectal cancer, highlighting the distinct characteristics associated with MMR deficiency. We observed a statistically significant association between MMR deficiency and younger age at diagnosis, female sex, right-sided tumor location, poor tumor differentiation, and loss of CDX2 expression. Among MMR proteins, MLH1 and PMS2 were most frequently affected, with concurrent loss being predominant.

These findings reinforce the value of MMR status as a biomarker not only for hereditary cancer screening and treatment stratification but also as a potential prognostic indicator. Despite certain limitations, particularly regarding the heterogeneity in immunohistochemical marker availability, our data support the integration of extended IHC panels and inflammatory markers into routine CRC evaluation. As a future direction, we propose validating these associations in larger, multicenter cohorts and developing integrated scoring systems combining inflammatory and immunohistochemical markers to support prognostic evaluation and treatment guidance in CRC.

## Figures and Tables

**Table 1:** Immunohistochemical markers evaluated in the study cohort.

Immunohistochemical Markers	Positive (Number of Patients)	Negative (Number of Patients)
MLH1, *n = 264*	220	43
MSH2, *n = 264*	256	8
MSH6, *n = 264*	259	5
PMS2, *n = 264*	225	39
CK7, *n = 20*	5	15
CK20, *n = 37*	33	4
CDX2, *n = 77*	65	12
Ki67, *n = 103*	Ki67 positivity rate (%)	Number of patients
	<25%	3
	>25%	100

**Table 2:** MMR deficiency in the study cohort.

Number of Patients n = 48	MLH1	MSH2	MSH6	PMS2
n = 1	√	√	√	√
n = 37	√			√
n = 4	√	√		
n = 3		√	√	
n = 1			√	
n = 1				√
n = 1	√			

**Table 3:** Comparative clinicopathological, immunohistochemical, and inflammatory profile according to MMR status in the study cohort.

Parameter		Positive MMR Expression n = 216	Negative MMR Expression n = 48	*p* Value
Age, n (%)	<50 years	29 (13.43%)	12 (25%)	0.853
	50–60 years	34 (15.74)	6 (12.5%)	
	60–70 years	71 (32.87%)	11 (22.92%)	
	70–80 years	65 (30.09%)	11 (22.92%)	
	≥80 years	17 (7.87%)	8 (16.66%)	
Gender, n (%)	Male	138 (63.9%)	18 (37.5%)	<0.001
	Female	78 (36.1%)	30 (62.55%)	
Tumor location, n (%)	Right colon	33 (15.28%)	26 (54.17%)	<0.001
	Left colon	93 (43.05%)	14 (29.17%)	
	Rectum	83 (38.43%)	6 (12.5%)	
	Synchronous tumors	7 (3.24%)	2 (4.16%)	
Endoscopic appearance, n (%)	Stenosing	124 (57.41%)	29 (60.42%)	0.917
	Exophytic	71 (32.87%)	15 (31.25%)	
	Infiltrative	21 (9.72%)	4 (8.33%)	
Histological type, n (%)	Adenocarcinoma NOS	193 (89.35%)	39 (81.25%)	0.225
	Mucinous adenocarcinoma	20 (9.26%)	7 (14.58%)	
	Others	3 (1.39%)	2 (4.17%)	
Tumor grade, n (%)	G1	14 (6.48%)	0 (0.00%)	<0.001
	G2	143 (66.2%)	18 (37.5%)	
	G3	59 (27.32%)	30 (62.5%)	
Tumor stage, n (%)	Stage I	31 (14.35%)	2 (4.17%)	0.216
	Stage II	89 (41.21%)	19 (39.58%)	
	Stage III	75 (34.72%)	21 (43.75%)	
	Stage IV	21 (9.72%)	6 (12.5%)	
Smoking, n (%)		26 (12.04%)	18 (37.5%)	<0.001
Alcohol, n (%)		21 (9.72%)	8 (16.67%)	0.266
Obesity, n (%)		56 (25.93%)	14 (29.17%)	0.780
Hypertension, n (%)		128 (59.26%)	28 (58.33%)	0.907
Atrial fibrillation, n (%)		22 (10.19%)	5 (10.42%)	0.971
Heart failure, n (%)		49 (22.69%)	13 (27.08%)	0.644
Chronic coronary syndrome, n (%)		41 (18.98%)	7 (14.58%)	0.477
Dyslipidemia, n (%)		69 (31.94%)	17 (35.42%)	0.769
Diabetes mellitus, n (%)		40 (1.85%)	10 (2.08%)	0.713
Ki67 n (%)	<25%	3 (3.53%)	0 (0.00%)	1.000
	>25%	82 (96.47%)	18 (100%)	
CK7 n (%)	Positive	3 (23.08%)	2 (28.57%)	1.000
	Negative	10 (76.92%)	5 (71.43%)	
CK20 n (%)	Positive	24 (92.30%)	9 (81.81%)	0.567
	Negative	2 (7.7%)	2 (18.19%)	
CDX2 n (%)	Positive	53 (94.64%)	12 (57.14%)	<0.001
	Negative	3 (5.36%)	9 (42.86%)	
NLR, median (range), n = 264	3.535 (1.27–20.31)		3.585 (1.47–21.4)	0.9415
PLR, median (range), n = 264	199.5 (32.55–658.57)		214.6 (58–985.45)	0.1608

**Table 4:** Correlation between tumor location and histological type and between tumor location and differentiation grade.

Tumor Location	Histological Type	Expected Frequency	Tumor Grade	Expected Frequency
Right colon	Adenocarcinoma NOS	68.798	G1	5.487
	Mucinous adenocarcinoma	8.048	G2	47.032
	Signet-ring cell adenocarcinoma	1.298	G3	28.480
	Neuroendocrine tumor	2.855		
Left colon	Adenocarcinoma NOS	106.169	G1	8.4
	Mucinous adenocarcinoma	12.419	G2	72.0
	Signet-ring cell adenocarcinoma	2.003	G3	43.6
	Neuroendocrine tumor	4.407		
Rectum	Adenocarcinoma NOS	82.387	G1	6.503
	Mucinous adenocarcinoma	9.637	G2	55.741
	Signet-ring cell adenocarcinoma	1.554	G3	33.754
	Neuroendocrine tumor	3.419		
Synchronous tumors	Adenocarcinoma NOS	7.644	G1	0.609
	Mucinous adenocarcinoma	0.894	G2	5.225
	Signet-ring cell adenocarcinoma	0.144	G3	3.164
	Neuroendocrine tumor	0.317		
χ^2^ = 8.38 (df = 9) *p* = 0.4966 Cramér’s V = 0			χ^2^ = 46.02 (df = 6) *p* < 0.001 Cramér’s V = 0.255	

## Data Availability

The data that support the findings of this study are available from the corresponding author upon reasonable request.
